# Therapy Landscape in Patients with Metastatic HER2-Positive Breast Cancer: Data from the PRAEGNANT Real-World Breast Cancer Registry

**DOI:** 10.3390/cancers11010010

**Published:** 2018-12-21

**Authors:** Michael P. Lux, Naiba Nabieva, Andreas D. Hartkopf, Jens Huober, Bernhard Volz, Florin-Andrei Taran, Friedrich Overkamp, Hans-Christian Kolberg, Peyman Hadji, Hans Tesch, Lothar Häberle, Johannes Ettl, Diana Lüftner, Markus Wallwiener, Volkmar Müller, Matthias W. Beckmann, Erik Belleville, Pauline Wimberger, Carsten Hielscher, Matthias Geberth, Wolfgang Abenhardt, Christian Kurbacher, Rachel Wuerstlein, Christoph Thomssen, Michael Untch, Peter A. Fasching, Wolfgang Janni, Tanja N. Fehm, Diethelm Wallwiener, Andreas Schneeweiss, Sara Y. Brucker

**Affiliations:** 1 Department of Gynecology and Obstetrics, Erlangen University Hospital, Comprehensive Cancer Center Erlangen-EMN, Friedrich-Alexander University of Erlangen–Nuremberg, Universitätsstrasse 21–23, 91054 Erlangen, Germany; michael.lux@uk-erlangen.de (M.P.L.); Naiba.Nabieva@uk-erlangen.de (N.N.); bernhard.volz@uk-erlangen.de (B.V.); lothar.haeberle@uk-erlangen.de (L.H.); matthias.beckmann@uk-erlangen.de (M.W.B.); 2 Department of Obstetrics and Gynecology, University of Tübingen, 72076 Tübingen, Germany; andreas.hartkopf@uni-tuebingen.de (A.D.H.); florin-andrei.taran@med.uni-tuebingen.de (F.-A.T.); diethelm.wallwiener@med.uni-tuebingen.de (D.W.); sara.brucker@med.uni-tuebingen.de (S.Y.B.); 3 Department of Gynecology and Obstetrics, Ulm University Hospital, 89070 Ulm, Germany; jens.huober@uniklinik-ulm.de (J.H.); wolfgang.janni@uniklinik-ulm.de (W.J.); 4 Oncologianova GmbH, 45657 Recklinghausen, Germany; overkamp@oncoconsult.onmicrosoft.com; 5 Marienhospital Bottrop, 46236 Bottrop, Germany; hans-christian.kolberg@mhb-bottrop.de; 6 Department of Bone Oncology, Nordwest Hospital, 60488 Frankfurt, Germany; hadji.peyman@khnw.de; 7 Oncology Practice at Bethanien Hospital Frankfurt; 60389 Frankfurt, Germany; hans.tesch@chop-studien.de; 8 Department of Gynecology and Obstetrics, Biostatistics Unit, Erlangen University Hospital, 91054 Erlangen, Germany; 9 Department of Obstetrics and Gynecology, Klinikum rechts der Isar, Technical University of Munich, 81675 Munich, Germany; johannes.ettl@tum.de; 10 Charité University Hospital, Berlin, Campus Benjamin Franklin, Department of Hematology, Oncology and Tumour Immunology, 12203 Berlin, Germany; diana.lueftner@charite.de; 11 Department of Obstetrics and Gynecology, University of Heidelberg, 69120 Heidelberg, Germany; markus.wallwiener@gmail.com (M.W.); andreas.schneeweiss@med.uni-heidelberg.de (A.S.); 12 Department of Gynecology, Hamburg-Eppendorf University Medical Center, 20246 Hamburg, Germany; v.mueller@uke.de; 13 ClinSol GmbH & Co KG, 97074 Würzburg, Germany; belleville@clin-sol.com; 14 Department of Gynecology and Obstetrics, Dresden University Hospital, 01307 Dresden, Germany; pauline.wimberger@uniklinikum-dresden.de; 15 gSUND Gynäkologie Kompetenzzentrum Stralsund, 18435 Stralsund, Germany; hielscher@gyn-stralsund.de; 16 Gynäkologische Praxisklinik am Rosengarten, 68165 Mannheim, Germany; mail@mgeberth.de; 17 Medizinischen Versorgungszentrum Onkologie, Onkologie im Elisenhof, 80335 Munich, Germany; abenhardt@t-online.de; 18 Department of Gynecology and Obstetrics, Medizinisches Zentrum Bonn Friedensplatz, 53111 Bonn, Germany; kurbacher@web.de; 19 Department of Gynecology and Obstetrics, Breast Center and Comprehensive Cancer Center Munich, Munich University Hospital, 80337 Munich, Germany; rachel.wuerstlein@med.uni-muenchen.de; 20 Department of Gynecology, Martin Luther University of Halle-Wittenberg, 06120 Halle (Saale), Germany; christoph.thomssen@uk-halle.de; 21 Department of Gynecology and Obstetrics, Helios Clinics Berlin Buch, 13125 Berlin, Germany; michael.untch@helios-gesundheit.de; 22 Department of Gynecology and Obstetrics, Düsseldorf University Hospital, 40225 Düsseldorf, Germany; tanja.fehm@med.uni-duesseldorf.de; 23 National Center for Tumor Diseases and Department of Gynecology and Obstetrics, Heidelberg University Hospital, 69120 Heidelberg, Germany

**Keywords:** advanced breast cancer, metastatic, chemotherapy, antihormone therapy, HER2 c-erbB2, HER2/neu, trastuzumab, pertuzumab, T-DM1, lapatinib

## Abstract

This study presents comprehensive real-world data on the use of anti-human epidermal growth factor receptor 2 (HER2) therapies in patients with HER2-positive metastatic breast cancer (MBC). Specifically, it describes therapy patterns with trastuzumab (H), pertuzumab + trastuzumab (PH), lapatinib (L), and trastuzumab emtansine (T-DM1). The PRAEGNANT study is a real-time, real-world registry for MBC patients. All therapy lines are documented. This analysis describes the utilization of anti-HER2 therapies as well as therapy sequences. Among 1936 patients in PRAEGNANT, 451 were HER2-positive (23.3%). In the analysis set (417 patients), 53% of whom were included in PRAEGNANT in the first-line setting, 241 were treated with H, 237 with PH, 85 with L, and 125 with T-DM1 during the course of their therapies. The sequence PH → T-DM1 was administered in 51 patients. Higher Eastern Cooperative Oncology Group (ECOG) scores, negative hormone receptor status, and visceral or brain metastases were associated with more frequent use of this therapy sequence. Most patients received T-DM1 after treatment with pertuzumab. Both novel therapies (PH and T-DM1) are utilized in a high proportion of HER2-positive breast cancer patients. As most patients receive T-DM1 after PH, real-world data may help to clarify whether the efficacy of this sequence is similar to that in the approval study.

## 1. Introduction

Overexpression of human epidermal growth factor receptor 2 (HER2), or amplification of the *HER2* gene, is seen in approximately 15–25% of breast cancer (BC) patients [1]. Since the discovery in the late 1980s of HER2 amplifications and their prognostic relevance [2], treatment for HER2-positive BC in this subgroup of patients has greatly improved [3,4,5,6]. Adding the monoclonal anti-HER2 antibody trastuzumab to standard chemotherapy resulted in a significant improvement in the progression-free survival (PFS) and overall survival (OS) in patients with metastatic HER2-positive BC [7]. These results led to the approval of trastuzumab for the treatment of HER2-positive metastatic BC.

Later, the dual tyrosine kinase inhibitor lapatinib was also analyzed in this group of patients. Women whose cancers had progressed after treatment with an anthracycline, a taxane, and trastuzumab were randomly assigned to therapy with capecitabine plus lapatinib or capecitabine alone. In contrast to the monotherapy, the combination treatment led to a significantly longer PFS. Therefore, lapatinib also became the standard of care in the early 2000s [8,9].

The CLEOPATRA study demonstrated an additional improvement in survival outcomes in treatment-naïve (chemotherapy and biological therapy, one endocrine treatment was allowed) HER2-positive patients with metastatic BC. Patients who were receiving docetaxel and dual HER2 blockade with trastuzumab plus pertuzumab, another monoclonal HER2 antibody, were compared with patients receiving docetaxel plus trastuzumab alone. The improved survival results led to the approval of pertuzumab for the first-line treatment setting. The enrolled patients were allowed to have had (neo)adjuvant chemotherapy with or without trastuzumab. However, the observed benefit of the addition of palliative pertuzumab was independent of any previous (neo)adjuvant treatment with trastuzumab [10,11].

Another HER2-targeted approved drug is trastuzumab emtansine (T-DM1), which was designed as an antibody–drug conjugate to target specifically HER2-enriched tumor cells, and in this way, reduce side effects in nontargeted tissue. In the EMILIA trial, the efficacy of T-DM1 was analyzed in women with HER2-positive advanced or metastatic disease who had previously been treated with a taxane and trastuzumab in the advanced therapy setting and were randomly assigned to second-line or further treatment with T-DM1 versus capecitabine plus lapatinib. It was found that T-DM1 was clearly superior with regard to survival outcomes in comparison with the control arm [12].

After the approval of T-DM1 and pertuzumab, the question arose of whether a combination of the two might result in an additional benefit. However, the MARIANNE study showed in first-line HER2-positive metastatic BC patients that neither T-DM1 alone nor T-DM1 in combination with pertuzumab improved the PFS in comparison with trastuzumab plus a taxane [13]. Although the reason for this remains unclear, there are cell line data that suggest that the correct therapy sequence for the drugs might have an influence on the treatment response [14].

Moreover, novel substances are also being investigated to further improve outcomes for patients. For instance, neratinib, another tyrosine kinase inhibitor, was recently approved for the adjuvant treatment of patients with HER2-positive early BC, due to its significant improvement of five-year disease-free survival (DFS) [15], and is currently also being analyzed in the metastatic setting. Afatinib, however, did not show any improvement in the outcomes for patients with metastatic BC in comparison with trastuzumab [16]. Margetuximab has now made available a third novel HER2 antibody that appears to enhance antibody-dependent cellular toxicity (ADCC), while at the same time being well-tolerated [17]. Its efficacy and safety are currently being investigated in the phase III SOPHIA trial in patients with HER2-positive metastatic BC who were previously treated with trastuzumab, pertuzumab, and T-DM1 [14].

As more and more therapy options become available, it is possible that treatment sequences may no longer be following the same inclusion and exclusion criteria as those that applied in the respective clinical trials. Understanding current therapy practice may be helpful for estimating the extent to which results from clinical trials can be generalized for specific patient populations. Therefore, the objective of this study is to describe comprehensive real-world evidence on the use of trastuzumab, pertuzumab, lapatinib, and T-DM1 in first-line treatment in the metastatic setting.

## 2. Patients and Methods

### 2.1. The PRAEGNANT Research Network

The PRAEGNANT study (Prospective Academic Translational Research Network for the Optimization of the Oncological Health Care Quality in the Adjuvant and Advanced/Metastatic Setting; NCT02338167 [18]) is an ongoing, prospective BC registry with a documentation system similar to that of a clinical trial. The aims of PRAEGNANT are to assess treatment patterns and quality of life, and to identify patients who may be eligible for clinical trials or specific targeted treatments [18,19,20,21]. Patients can be included at any point in time during the course of their disease. All of the patients included in the present study provided informed consent, and the study was approved by the relevant ethics committees.

### 2.2. Patients

**Figure 1:**
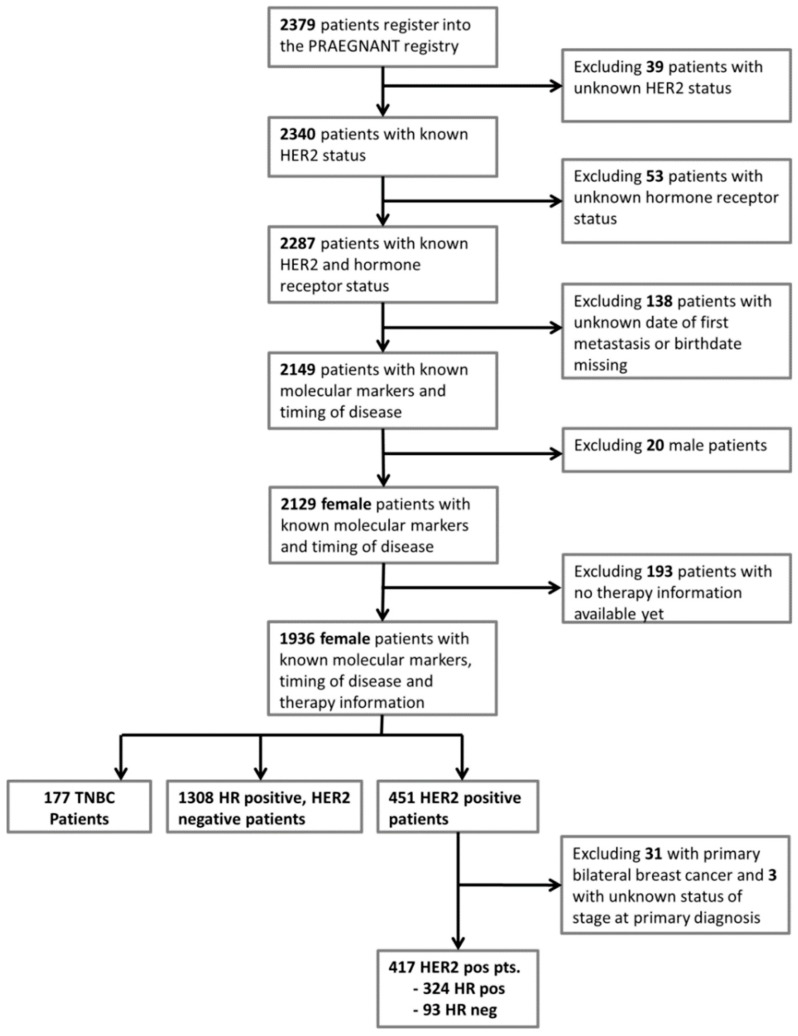
Patient flow chart and patient selection.

A total of 2379 patients with advanced or metastatic BC were registered in the PRAEGNANT study between July 2014 and March 2018 at 52 study sites. Patients were excluded in the following hierarchical order: 39 patients were excluded due to unknown HER2 status, as well as 53 patients due to unknown hormone receptor status. In 138 patients, the date of the first diagnosis of a metastasis or their birth date was missin. Therefore, these patients also had to be excluded. Male patients (*n* = 20) were also not included in the analysis. Treatment information was not available for an additional 193 women, leaving 1936 patients for whom the above-mentioned data were known. A total of 451 of these women had HER2-positive tumors (Figure 1). For analysis, patients were divided into distinct patient groups based on the documentation status concerning the therapy lines. Group 1 is defined as the patient population for which only the first therapy line is documented. Group 2 is the population for which the first and the second therapy lines are documented. Group 3 is the patient population for which only the first, seco, nd and third therapy lines are documented. Group 4 is the patient population for which at least the first to the forth therapy lines are documented. Each group cannot be part of the other groups. These groups are the natural consequence of patients being treated with more or less therapy lines in the metastatic setting.

### 2.3. Data Collection

The data were collected by trained staff and documented in an electronic case report form [18]. The data were monitored using automated plausibility checks and on-site monitoring. Data that are not usually documented as part of routine clinical work are collected prospectively using structured questionnaires completed on paper. These consist of epidemiological data, such as family history, cancer risk factors, quality of life, nutrition and lifestyle items, and psychological health. Supplementary Table S1 provides an overview of the collected data.

### 2.4. Definition of Hormone Receptors, HER2 Status and Grading

The definition of hormone receptors, HER2 status, and grading was described previously [19]. Briefly, data about estrogen receptor status, progesterone receptor status, HER2 status, and grading were obtained for documentation purposes for each tumor that had been biopsied. Therefore, there could be several possible sources (right breast, left breast, local recurrence, metastatic site). Biomarker status for ER, PR, and HER2 were determined as follows: If a biomarker assessment of the metastatic site was available, this receptor status was used for the analysis. If there was no information available for metastases, the latest biomarker results from the primary tumor were used. Additionally, all patients who received estrogen therapy in the metastatic setting were assumed to be HR-positive, and all patients who had ever received anti-HER2 therapy were assumed to be HER2-positive. There was no central review of biomarkers. The study protocol recommended assessing ER and PR status as positive if ≥ 1% was stained. A positive HER2 status required an immunohistochemistry score of 3+ or positive fluorescence in situ hybridization/competitive in situ hybridization (FISH/CISH).

### 2.5. Statistical Considerations

The analysis and reporting of treatments are descriptive. The total number of treatments for each of the following four therapy lines are provided: Trastuzumab (H), trastuzumab and pertuzumab (PH), lapatinib (L), and trastuzumab emtansine (T-DM1). It was also analyzed whether patients who had already completed a specific number of therapy lines (1–4) received these four anti-HER2 therapies in any therapy line. For this purpose, the patients were categorized into four distinct groups, namely: Patients for whom only the first therapy line was documented, patients for whom the first two therapy lines were documented, patients for whom the first three therapy lines were documented, and patients for whom at least the first four therapy lines were documented. Similarly, the frequencies of usage of the PH → T-DM1 and T-DM1 → PH therapy sequences were analyzed, regardless of whether these therapies followed each other directly.

It was also analyzed whether the patients’ characteristics were associated with the frequency of utilization of the PH → T-DM1 sequence in the first four therapy lines, again regardless of whether these therapies followed each other directly.

With regard to the year of therapy, the patients were also categorized in relation to their first four therapy lines. The first group consisted of patients who completed all documented therapy lines before 2013, the second group had to have had at least one treatment administered before 2013 and one in 2013 or after 2013. In the last group, all patients had to have received all treatments after 2013.

Calculations were performed using IBM SPSS Statistics, version 24 (Armonk, New York, NY, USA: IBM Corporation).

## 3. Results

### 3.1. Patient and Disease Characteristics

**Figure 2:**
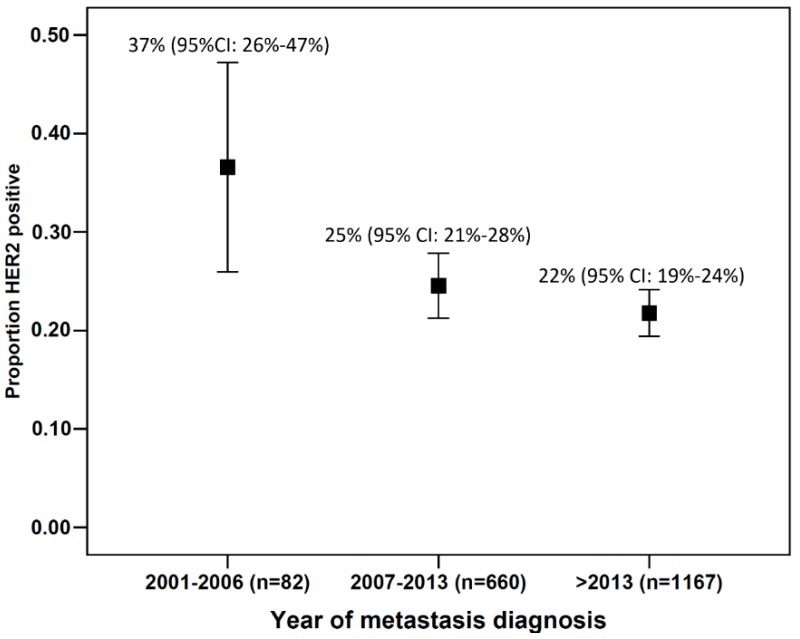
Proportion of human epidermal growth factor receptor 2 (HER2)-positive patients with 95% confidence intervals relative to the year in which the metastases were diagnosed.

A total of 451 (23.3%) patients in the registry had HER2-positive metastatic breast cancer. Figure 2 shows the frequency of HER2-positive metastatic breast cancer patients over the years. Although the HER2 status was positive in 37% (95% CI: 26–47%) of all patients with metastases who were treated up to 2006, HER2 positivity was seen in 25% (95% CI: 21–28%) and 22% (95% CI: 19–24%) of patients diagnosed with metastases in 2007–2013 and after 2013, respectively (Figure 2). For further analyses, patients with bilateral breast cancer at diagnosis and those with missing information about the stage at initial diagnosis were excluded. The final study population comprised 417 patients, 324 of whom were hormone receptor–positive and 93 hormone receptor–negative (Figure 1).

The characteristics of the patients and diseases are listed in Table 1. Most patients entered the study in the first-line setting, had an Eastern Cooperative Oncology Group (ECOG) score of 0, and had visceral metastases. Approximately 40% of the patients had metastases at the time of diagnosis.

### 3.2. Therapies

Across all of the therapy lines (1 to 9+), 241 of the 417 patients were treated with H without additional anti-HER2 therapy, 237 with PH, 85 with L, and 125 with T-DM1. The respective figures up to therapy line four are 236 (H), 220 (PH), 79 (L), and 108 (T-DM1) patients.

Table 2 shows patterns of therapy utilization relative to patient groups with first-line therapy documented, with first- and second-line therapy documented, with first- to third-line therapy documented, and with first- to fourth-line therapy documented, relative to the treatment period. Trastuzumab, either as a single anti-HER2 therapy or together with pertuzumab, was already administered in over 80% of the patients for whom only the first-line was documented. PH utilization increased across the different time periods, with approximately 60–70% of all patients already receiving this treatment as first-line therapy. T-DM1 utilization also increased across the time periods, although patients with a larger number of documented therapy lines had a higher frequency (approximately 52% of patients had four therapy lines documented and approximately 33% of patients had only two therapy lines documented). Lapatinib use did not change across the time periods and was mainly administered in later therapy lines (Table 2).

The sequence of PH followed by T-DM1 (PH → T-DM1) was administered in 51 patients throughout all therapy lines and in 50 patients in lines one to four. Of those 50 patients, 48 patients were treated with the combination of PH and chemotherapy, and two with the combination of PH and endocrine therapy. Eleven patients received a sequence of T-DM1 → PH, eight of whom were treated within the first four therapy lines. This is equivalent to a utilization rate of PH → T-DM1 up to therapy line four after approval of about 42%, with an increase of approximately 10% from lines two to four per therapy line (Table 3).

### 3.3. Predictors of the Use of a Therapy Sequence of PH Followed by T-DM1

Several patient and disease characteristics were analyzed in relation to their influence on the utilization of the therapy sequence PH → T-DM1 (Table 4). Age, grading, and stage at the initial diagnosis did not have any influence. Patients with higher ECOG scores appeared to be treated with this sequence more often, as well as patients with brain or visceral metastases. One patient with metastases only in the bone was treated with PH → T-DM1. Patients with a positive hormone receptor status were less frequently treated with the PH → T-DM1 sequence. Although only 23.7% of patients with a positive hormone receptor status received this sequence up to therapy line four, hormone receptor–negative patients were treated with this sequence in about 44% of cases.

## 4. Discussion

This analysis of a cohort from a real-world breast cancer registry shows how frequently anti-HER2 therapies are used. Although most patients received trastuzumab, the percentage of patients who received pertuzumab and trastuzumab, lapatinib, or T-DM1 was clearly lower. Most of the trastuzumab and pertuzumab therapies were administered in the first-line setting, but TDM-1 was administered in most cases between the second and fourth lines, and lapatinib more often in the third- and fourth-line setting. The sequence of TDM-1 after trastuzumab and pertuzumab was administered in up to 40% of patients with four therapy lines, while the sequence T-DM1 followed by pertuzumab was only administered in about 5% of the patients.

The analysis shows that HER2-positive metastatic breast cancer is a subgroup with a clinically relevant frequency. The frequency of HER2-positive patients in the present cohort of metastatic breast cancer patients was 23.3%, a rate similar to the initially described frequencies of 25–30% in primary breast cancer before the introduction of anti-HER2 therapies [2,22]. The frequency of triple-negative breast cancer was much lower in this cohort, at 9.1% of all cases. Looking at HER2 positivity over the years, it seems that HER2 positivity decreased over time. One possible explanation could be the introduction of trastuzumab in the adjuvant setting [23,24,25], possibly reducing the number of HER2 positive patients, who would develop a metastasis at a later timepoint. However, it should be noted that the PRAEGNANT study has been registering patients since 2014, and data from before that year are purely descriptive. Therefore, this trend might be the result of a bias.

Pertuzumab and T-DM1 were approved in Germany in 2013. These therapies were thus inevitably not prevalent in the cohort before that time. Few patients were treated in clinical trials before that, and it can be clearly seen that the use of pertuzumab and trastuzumab during the first four therapy lines increased from 27–36% around 2013 to 63–71% after 2013. Most of these treatments were administered as first-line therapy, which is in accordance with the current national therapy guidelines [26]. T-DM1, which is administered after tumor progression in the metastatic setting, was already used in 23–58% of patients around 2013 and continued to be administered in 33–53% of patients after 2013. This therapy pattern also matched the current national therapy guidelines [26]. Most patients received T-DM1 after pertuzumab—a sequence that is under discussion, since at the time when the EMILIA study was conducted, only previous trastuzumab therapy was available [12]. A recent retrospective study did not show any differences in the prognosis when patients who had been treated with T-DM1 after pertuzumab were compared with patients who had not received previous T-DM1 treatment [27]. However, analyses of differences between subgroups with earlier and later therapy lines in which T-DM1 was administered were inconclusive [27]. The use of lapatinib did not change much over time in the first four therapy lines, despite the introduction of pertuzumab and T-DM1.

With regard to possible predictive factors that may have influenced physicians in deciding to treat patients with the pertuzumab–trastuzumab sequence, it appears that patients with more advanced disease or a less favorable prognosis were more likely to be treated with the PH → T-DM1 therapy sequence. Parameters that were associated with a higher frequency of PH → T-DM1 use were poorer ECOG scores, brain and visceral metastases, negative hormone receptor status, and higher grading. The higher frequency of PH → T-DM1 therapy in patients with brain metastases could be the enrichment of patients with brain metastases in patients treated with anti-HER2 therapies, while in patients with positive hormone receptor status, the avoidance of chemotherapy could be a motivation behind not giving an anti-HER2 directed therapy. With regard to visceral metastasis, its more frequent use in patients with visceral metastases could be explained with the possible need for an effective therapy regimen including anti-HER2 treatments and chemotherapy. Moreover, in exploratory subgroup analyses of both studies, CLEOPATRA and EMILIA patients with visceral metastases had a larger benefit from PH or T-DM1 than the patients treated with the respective comparator therapies [10,11,12].

To the best of our knowledge, no comparable data concerning this healthcare research question was previously published. Hormone receptor status in particular appears to be of special interest, since a desire to avoid chemotherapy in this patient group is a possible reason why specific treatment regimens are not administered.

This study has several strengths and limitations. Although the PRAEGNANT breast cancer registry has registered more than 2300 patients with metastatic breast cancer, only 451 of the patients were HER2-positive. Although this is a large number in comparison with other publications reporting on prospective or retrospective cohorts, the sample size might be low for identifying treatment patterns and possible predictors of patient/tumor characteristics that are associated with specific therapy sequences and possible outcomes. Clinical cancer registries might be helpful for gathering data on larger patient populations [28], but the degree of detail in the information might be limited in such population-based registries. Data completeness and detail are certainly strengths in the PRAEGNANT registry, which documents therapy lines, side effects, progression, and mortality with an approach similar to that used in clinical trials. Another fact that needs to be taken into consideration when interpreting the data is that in real-world cohorts, not all patients enter the study or end the study at comparable time points. Patients in the first-line setting may therefore be overrepresented, as patients die during the course of the disease, or may be lost to follow-up. An attempt was made to account for this by categorizing the patients into groups for which documentation for all therapy lines up to a specific line was available, with treatment utilization being reported for each of these groups separately.

## 5. Conclusions

In conclusion, the utilization of trastuzumab appeared to be sufficiently high in this cohort of patients with metastatic breast cancer. The utilization of the PH → T-DM1 sequence appeared to be rather low, and the reasons for this should be analyzed in future studies.

## Figures and Tables

**Table 1:** Patient characteristics at baseline.

Characteristic	n or Mean	% or SD
Age at study entry	57.9	13.0
BMI	26.0	5.3
Time from diagnosis to metastasis (days)	1177.8	1743.0
Therapy situation at study entry		
First-line	223	53.5
Second-line	70	16.8
Third-line	53	12.7
Fourth-line	26	6.2
Fifth-line and higher	34	8.2
Therapy situation at database closure		
First-line	171	41.0
Second-line	82	19.7
Third-line	47	11.3
Fourth-line	17	4.1
Fifth-line and higher	59	14.1
Hormone receptor status		
Negative	93	22.3
Positive	324	77.7
ECOG		
0	196	47.0
1	155	37.2
2	35	8.4
3	12	2.9
4	2	0. 4
Metastasis site at study entry		
Brain ^a^	79	18.9
Visceral ^b^	222	53.2
Bone only	58	13.9
Other ^c^	50	12.0
Metastatic at time of diagnosis		
No	244	58.5
Yes	173	41.5

BMI (body mass index) ECOG (Eastern Cooperative Oncology Group) (score); SD (standard deviation). ^a^ Patients included in the “brain” group were allowed to have metastases at any other site. ^b^ Patients included in the “visceral” group were allowed to have metastases at any other site except the brain. ^c^ Patients included in this group were not allowed to have any brain, visceral, or bone metastases.

**Table 2:** Frequencies of patients who received the respective treatments. The patients are categorized here into four mutually exclusive (distinct) patient groups according to the number of documented therapy lines. The percentages of patients treated are marked in bold. The numbers and percentages of treated patients refer to the cumulative number of patients treated up to the highest documented therapy line. For example, in the group of patients with three therapy lines documented and treated after 2013, 33 patients have been treated with trastuzumab in one of the first three therapy lines.

Therapy	Patients Treated Before 2012		Patients Treated Crossing 2013		Patients Treated After 2013	
	Not Treated	Treated	Not Treated	Treated	Not Treated	Treated
Trastuzumab (H)						
treatments in patient group 1 ^a^	4 (19)	17 (**80.9**)	0 (0)	6 (**100**)	28 (20.4)	109 (**79.5**)
treatments in patient group 2 ^b^	0 (0)	6 (**100**)	2 (13.3)	13 (**86.6**)	11 (15)	62 (**84.9**)
treatments in patient group 3 ^c^	0 (0)	4 (**100**)	3 (15.7)	16 (**84.2**)	6 (15.3)	33 (**84.6**)
treatments in patient group 4 ^d^	3 (20)	12 (**80**)	8 (18.1)	36 (**81.8**)	7 (18.4)	31 (**81.5**)
Trastuzumab + pertuzumab (PH)						
treatments in patient group 1 ^a^	19 (90.4)	2 (**9.5**)	3 (50)	3 (**50**)	51 (37.2)	86 (**62.7**)
treatments in patient group 2 ^b^	6 (100)	0 (**0**)	11 (73.3)	4 (**26.6**)	21 (28.7)	52 (**71.2**)
treatments in patient group 3 ^c^	4 (100)	0 (**0**)	13 (68.4)	6 (**31.5**)	12 (30.7)	27 (**69.2**)
treatments in patient group 4 ^d^	15 (100)	0 (**0**)	28 (63.6)	16 (**36.3**)	14 (36.8)	24 (**63.1**)
Lapatinib (L)						
treatments in patient group 1 ^a^	20 (95.2)	1 (**4.7**)	5 (83.3)	1 (**16.6**)	134 (97.8)	3 **(2.1**)
treatments in patient group 2 ^b^	6 (100)	0 (**0**)	12 (80)	3 (**20**)	65 (89)	8 (**10.9**)
treatments in patient group 3 ^c^	3 (75)	1 (**25**)	15 (78.9)	4 (**21**)	30 (76.9)	9 (**23**)
treatments in patient group 4 ^d^	8 (53.3)	7 (**46.6**)	22 (50)	22 (**50**)	18 (47.3)	20 (**52.6**)
Trastuzumab emtansine (T-DM1)						
treatments in patient group 1 ^a^	21 (100)	0 (**0**)	6 (100)	0 (**0**)	131 (95.6)	6 (**4.3**)
treatments in patient group 2 ^b^	6 (100)	0 (**0**)	10 (66.6)	5 (**33.3**)	49 (67.1)	24 (**32.8**)
treatments in patient group 3 ^c^	4 (100)	0 (**0**)	8 (42.1)	11 (**57.8**)	21 (53.8)	18 (**46.1**)
treatments in patient group 4 ^d^	14 (93.3)	1 (**6.6**)	21 (47.7)	23 (**52.2**)	18 (47.3)	20 (**52.6**)

^a^ Group 1 is the patient population for which only the 1st therapy line is documented. These patients are not part of groups 2–4; ^b^ Group 2 is the patient population for which only the 1st and the 2nd therapy lines are documented. These patients are not part of the other groups. ^c^ Group 3 is the patient population for which only the 1st, 2nd and 3rd therapy lines are documented. These patients are not part of the other groups. ^d^ Group 4 is the patient population for which the 1st to the 4th therapy lines are documented. These patients are not part of the other groups.

**Table 3:** Frequencies of patients who were treated with the respective treatment sequences, irrespective of whether the sequences were administered directly after each other. The patients are categorized into four mutually exclusive patient groups according to the numbers of documented therapy lines. The percentages of patients treated are marked in bold.

Therapy	Patients Treated Before 2012		Patients Treated Crossing 2012		Patients Treated After 2012	
	Not Treated	Treated	Not Treated	Treated	Not Treated	Treated
Pertuzumab/trastuzumab → trastuzumab emtansine (PH → T-DM1)						
treatments in patient group 1 ^a^	21 (100)	0 (**0**)	6 (100)	0 (**0**)	137 (100)	0 (**0**)
treatments in patient group 2 ^b^	6 (100)	0 (**0**)	14 (93.3)	1 (**6.6**)	59 (80.8)	14 (**19.1**)
treatments in patient group 3 ^c^	4 (100)	0 (**0**)	17 (89.4)	2 (**10.5**)	27 (69.2)	12 (**30.7**)
treatments in patient group 4 ^d^	15 (100)	0 (**0**)	39 (88.6)	5 (**11.3**)	22 (57.8)	16 (**42.1**)
Trastuzumab emtansine → pertuzumab/trastuzumab (T-DM1 → PH)						
treatments in patient group 1 ^a^	21 (100)	0 (**0**)	6 (100)	0 (**0**)	136 (99.2)	1 (**0.7**)
treatments in patient group 2 ^b^	6 (100)	0 (**0**)	15 (100)	0 (**0**)	73 (100)	0 (**0**)
treatments in patient group 3 ^c^	4 (100)	0 (**0**)	18 (94.7)	1 (**5.2**)	38 (97.4)	1 (**2.5**)
treatments in patient group 4 ^d^	15 (100)	0 (**0**)	41 (93.1)	3 (**6.8**)	36 (94.7)	2 (**5.2**)

^a^ Group 1 is the patient population for which only the 1st therapy line is documented. These patients are not part of groups 2–4; ^b^ Group 2 is the patient population for which only the 1st and the 2nd therapy lines are documented. These patients are not part of the other groups. ^c^ Group 3 is the patient population for which only the 1st, 2nd and 3rd therapy lines are documented. These patients are not part of the other groups. ^d^ Group 4 is the patient population for which the 1st to the 4th therapy lines are documented. These patients are not part of the other groups.

**Table 4:** Frequency of patients who received the treatment sequence pertuzumab/trastuzumab → trastuzumab emtansine (PH → T-DM1), irrespective of whether the sequences were administered directly after each other. All patients who had at least two documented therapy lines and in whom all treatments started after 2013 are included.

Characteristic	PH → T-DM1	
	No	Yes
Age		
<50	28 (65.1)	15 (34.9)
50–65	53 (72.6)	20 (27.4)
>65	27 (79.4)	7 (20.6)
Eastern Cooperative Oncology Group (ECOG) score		
0	59 (79.7)	15 (20.3)
1	34 (63.0)	20 (37.0)
2	6 (60.0)	4 (40.0)
3	5 (100)	0 (0)
Metastasis site at study entry		
Brain ^a^	14 (58.3)	10 (41.7)
Visceral ^b^	56 (68.3)	23 (31.7)
Bone only ^c^	16 (94.1)	1 (5.9)
Other ^d^	19 (82.6)	4 (14.7)
Hormone receptor status		
Negative	18 (56.3)	14 (43.8)
Positive	90 (76.3)	28 (23.7)
Grade		
1	2 (100)	0 (0)
2	45 (78.9)	12 (21.1)
3	50 (64.1)	28 (35.9)
Primary metastatic		
No	68 (73.9)	24 (26.1)
Yes	40 (69.0)	42 (28.0)

^a^ Patients included in the “brain” group were allowed to have metastases at any other site. ^b^ Patients included in the “visceral” group were allowed to have metastases at any other site except the brain. ^c^ Patients included in this group were not allowed to have any brain, visceral, or bone metastases.

## Supplementary Materials

The following are available online at http://www.mdpi.com/2072-6694/11/1/10/s1, Table S1: Data categories captured in the PRAEGNANT study.
