# Supplementary material for: Therapy Landscape in Patients with Metastatic HER2-Positive Breast Cancer: Data from the PRAEGNANT Real-World Breast Cancer Registry
Source: Cancers (Basel). 2018 Dec 21;11(1):10. doi: 10.3390/cancers11010010 (PMC6357172; doi:10.3390/cancers11010010)
Supplement: Supplementary file 1 [file cancers-11-00010-s001.pdf]

cancers

Supplementary Materials: Therapy Landscape in Patients with Metastatic HER2-Positive Breast Cancer: Data from the PRAEGNANT Real-World Breast Cancer Registry

Michael P. Lux, Naiba Nabieva, Andreas D. Hartkopf, Jens Huober, Bernhard Volz, Florin-Andrei Taran, Friedrich Overkamp, Hans-Christian Kolberg, Peyman Hadji, Hans Tesch, Lothar Häberle, Johannes Ettl, Diana Lüftner, Markus Wallwiener, Volkmar Müller, Matthias W. Beckmann, Erik Belleville, Pauline Wimberger, Carsten Hielscher, Matthias Geberth, Wolfgang Abenhardt, Christian Kurbacher, Rachel Wuerstlein, Christoph Thomssen, Michael Untch, Peter A. Fasching, Wolfgang Janni, Tanja N. Fehm, Diethelm Wallwiener, Andreas Schneeweiss and Sara Y. Brucker

Supplementary Table S1. Data categories captured in the PRAEGNANT study.

Data Continuously Captured, if Applicable	Data Assessed at Study Entry	Data Assessed at Follow-Up Care Appointments
Concomitant diseases	Life status, ECOG	Life status, ECOG
Concomitant medication	Quality of life	Quality of life
Cancer systemic therapies	Breast cancer risk factor questionnaire	Breast and axilla evaluation
Cancer radiotherapy	Breast and axilla evaluation	Distant metastasis evaluation
Cancer surgery	Distant metastasis evaluation	Biomaterial ascertainment
Breast cancer right side	Biomaterial ascertainment	PRO questionnaires
Breast cancer left side	PRO questionnaires	

ECOG, Eastern Cooperative Oncology Group (performance status); PRO, patient-reported outcome.
